# The Impact of Chinese Adult’s Food Literacy on Healthy Eating Intentions Based on the Planned Behaviour Theory

**DOI:** 10.3390/nu17203295

**Published:** 2025-10-20

**Authors:** Yingying Li, Ji-Yun Hwang

**Affiliations:** 1 Department of Foodservice Management and Nutrition, Graduate School, Sangmyung University, Seoul 03016, Republic of Korea; liy407853@gmail.com; 2 Major of Foodservice Management and Nutrition, Sangmyung University, Seoul 03016, Republic of Korea

**Keywords:** food literacy, healthy eating habits, behavioural intentions, theory of planned behaviour, adults, China

## Abstract

Background: Unhealthy diets are major contributors to obesity and chronic diseases. In 2023, 50.7% of Chinese adults were overweight or obese, underscoring the need to strengthen healthy-eating intentions. Methods: We analysed a cross-sectional online survey of 1145 adults (18–64 years) from Henan and Shandong. Moderation was tested using multiple linear regression with mean-centred interaction terms between each Theory of Planned Behaviour (TPB) construct (attitude, subjective norms, and perceived behavioural control [PBC]) and each food-literacy component (production, choices, preparation and cooking, intake, disposal). Models were adjusted for age, occupation, marital status, alcohol use, physician-diagnosed chronic disease, and living with family. To address multicollinearity, we performed a ridge-regression robustness check (L2-regularised linear model; λ = 0.02 selected by 10-fold cross-validation; CV-RMSE = 0.483; CV-R^2^ = 0.631). We report B, SE, β, *p*-values, and R^2^/adjusted R^2^. Results: The overall food-literacy score did not significantly moderate the associations between attitude, subjective norms, or PBC and healthy-eating intention (*p* = 0.328, 0.671, 0.985). In component-wise analyses, only intake (intake) significantly moderated the PBC–intention association (B = 0.002, SE = 0.001, t = 2.497, *p* = 0.013); in the ridge model, the effect remained positive (β = 0.182; λ = 0.02). PBC (β = 0.459) and subjective norms (β = 0.169) were the strongest main-effect predictors. The best-fitting model explained R^2^ = 0.663 of the variance in intention (adjusted R^2^ = 0.663). Conclusions: Among adults in Henan and Shandong, the intake component of food literacy strengthened the association between PBC and healthy-eating intention, whereas overall food literacy showed no general moderating effect. Interventions should prioritise intake-related skills (e.g., portion planning, lower-sodium choices and nutrition label use) to enhance perceived behavioural control and, in turn, intention. Given the cross-sectional design, causal inference is limited; longitudinal, capability-building evaluations are warranted.

## 1. Introduction

Since 1978, when China launched its national “reform and opening-up” program of market-oriented economic reforms, agricultural output—including grain production—has increased substantially [1]. However, ensuring optimal nutritional health among residents continues to present substantial challenges. Inappropriate dietary habits have contributed to a growing incidence of chronic diseases, particularly cardiovascular diseases, stroke, and type 2 diabetes [2,3,4]. Insufficient intake of whole grains, vegetables, and fruits, combined with high salt intake, is considered a major cause of these conditions [1,5,6,7]. According to the Scientific Research Report on Dietary Guidelines for Chinese Residents (2021), excessive sodium intake, along with the rapidly increasing intake of red meat, is among the main dietary factors contributing to cardiovascular and metabolic mortality [8]. Obesity and diabetes have become increasingly severe public health concerns. In 2023, the prevalence of overweight and obesity among Chinese adults aged 18 years and above reached 50.7%, with 34.3% classified as overweight and 16.4% as obese [9]. In recent years, excess body weight has been strongly associated with rising rates of insulin resistance and diabetes among Chinese adults, with high body mass index (BMI) identified as the primary individual risk factor for diabetes in China [10,11,12,13].

With the rapid expansion of the national economy, advances in communication technologies, and the rise of platform-based economies, residents’ dietary patterns have shifted toward more convenience-oriented options. Online food delivery has become integral to daily intake—by December 2023, there were 545 million users nationwide (49.9% of all internet users) [14]. These structural changes, together with the high prevalence of overweight and obesity among adults in China (50.7% in 2023) [9], underscore growing public-health concerns and motivate the present focus on food literacy within the TPB framework. In this context, the significance of food literacy has become increasingly apparent.

Food literacy represents a crucial dimension of overall health literacy and plays a key role in improving dietary patterns and nutritional health among Chinese residents. The ability to comprehend food-related information determines not only how individuals choose and consume food, but also how effectively they manage, prepare, and cook it [15]. Although research on food literacy in China has expanded in recent years, it still lags behind that of developed countries. Notably, levels among young people remain relatively low; for example, a 2021 study in Shenzhen reported that only 19.52% of youths met adequacy on a food- and nutrition-literacy scale [16]. Food literacy is a key dimension of health literacy that shapes dietary patterns and nutritional health. Although definitions vary across the literature [15,17,18,19,20,21,22,23,24,25,26,27,28,29,30,31,32,33,34,35,36], we follow a widely cited international definition—that of Vidgen and Gallegos (2014)—which views food literacy as the interrelated knowledge, skills, and behaviours needed to plan, manage, select, prepare, and eat food to meet needs [15]. Aligning with this perspective and with our measurement tool, we operationalize food literacy across five food-system domains—production, distribution, selection, preparation and cooking, intake, and waste disposal—capturing decision making along the “farm to table” continuum and emphasizing actionable knowledge and skills. This operationalization and scoring approach are grounded in the validated instrument we draw upon (adult form; 0–4 Likert items; domain scores aggregated to a total score) and its conceptual framework of food literacy covering production to disposal. We therefore use the single term “food literacy” throughout, reserving original labels only when referring to specific instrument names [37].

The Theory of Planned Behaviour (TPB) explains intention through attitudes, subjective norms, and perceived behavioural control (PBC) (Ajzen, 1985) [38]. In 1991, Ajzen reformulated the model to emphasise the proximal relations among these constructs and behavioural intention. The revised TPB has been widely applied across health, consumer, and environmental domains [38,39,40], and numerous studies support its explanatory value for health-related intentions and consumer decision making [41,42]. Ajzen et al. (2011) further highlighted the role of accurate domain knowledge in shaping beliefs and, ultimately, intention within the TPB framework [43]. Building on this insight, Ölander and Thøgersen’s Motivation–Opportunity–Capability (MOC) model conceptualises capability—the knowledge and skills required to perform a behaviour—as both a direct determinant of behaviour and a moderator that can condition TPB pathways [44]. In dietary contexts, food literacy can be treated as a behavioural capability by improving the knowledge and skills needed to plan, select, prepare, and consume healthier options, it enhances perceived behavioural control and lowers execution barriers (e.g., time constraints and label comprehension), thereby strengthening healthy-eating intentions [45].

This study integrates food literacy into the TPB to examine how food literacy influences individuals’ intentions towards healthy eating. However, in the Chinese context there is limited empirical work that explicitly integrates food literacy—conceptualised as a behavioural capability—into the TPB and tests its moderating role; evidence that compares the five components of food literacy is particularly sparse, especially in regional adult samples. Therefore, this study aims to examine whether food literacy and its components moderate the relationships between TPB constructs (attitudes, subjective norms and perceived behavioural control) and healthy-eating intentions among Chinese adults, with data from Henan and Shandong. Specifically, we ask(1) does overall food literacy moderate the associations between TPB constructs and healthy-eating intention? and (2) do the five components (production, choices, preparation and cooking, intake and disposal) differentially moderate these associations?

## 2. Materials and Methods

### 2.1. Settings and Participants

The study protocol has been described in detail elsewhere [46,47,48,49]. Briefly, we conducted two waves of online surveys in 2023–2024 among adults residing in selected areas of the Henan and Shandong Provinces, China. Data collection was led by Chinese investigators in their home regions; the survey was minimal risk and approved by the PI’s home IRB (Sangmyung University, Seoul, Republic of Korea), with instruments translated, culturally adapted, and piloted for Chinese adults. Using a snowball sampling approach, questionnaires were distributed via QR codes and WeChat links. A total of 1252 Chinese adults completed the survey, which included an incentive. To ensure data quality, we applied five pre-specified screening criteria and excluded 107 respondents, yielding a final analytic sample of 1145 eligible participants. The study received institutional review board approval from Sangmyung University (IRB approval numbers: SMUIRBC-2023-001 [23 June 2023] and SMUIRBC-2024-024 [12 June 2024]).

### 2.2. Conceptual Framework

**Figure 1:**
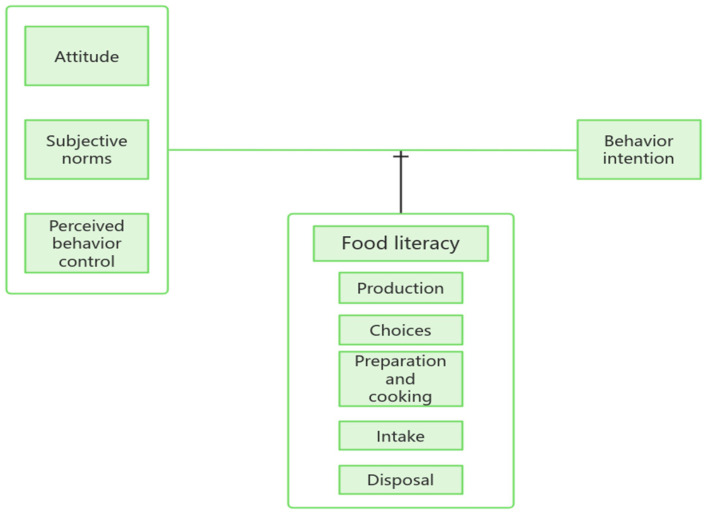
Study conceptual framework.

This study explored the impact of food literacy on the relationship between the three main components of planned behaviour theory (i.e., attitude, subjective norms, and perceived behaviour control) and behavioural intent (Figure 1). The research assumptions under the conceptual framework are as follows: First, attitudes, subjective norms, and perceptual behaviour control have a positive impact on healthy behavioural intentions. Second, food literacy has a positive impact on the relationship among attitude, subjective norms, PBC, and behavioural intentions. Third, the five components of food literacy (i.e., production, choices, preparation and cooking, intake, and disposal) have a positive impact on the relationship among attitude, subjective norms, perceptual behaviour control, and behavioural intent.

**Hypotheses** **(H1):** *Attitudes, subjective norms, and perceived behavioural control (PBC) will be positively associated with healthy-eating intention, with PBC expected to be the strongest predictor based on prior TPB evidence.*

**Hypotheses** **(H2):** *Overall food literacy will moderate the associations between TPB constructs and intention (i.e., stronger TPB–intention relations at higher rates of literacy).*

Exploratory analyses: Given limited prior evidence at the domain level, we explored whether any of the five food-literacy domains (production, choices, preparation and cooking, consumption, and waste disposal) moderated the TPB–intention pathways without specifying a particular domain a priori.

### 2.3. Survey Instrument

#### 2.3.1. TPB

According to the TPB, behavioural intention is determined by three core constructs: attitude, subjective norms, and perceived behavioural control (PBC). The development of the present questionnaire has been reported in detail elsewhere [46,47,48,49]. In brief, an expert panel adapted the instrument for Chinese adults through forward translation, review, and refinement, followed by a pilot test with nutrition-major students to ensure clarity and cultural appropriateness. To enhance content validity and coverage, we also drew on instruments previously validated in adolescent populations [50,51]. Our earlier work indicates that the instrument exhibits strong explanatory performance among Chinese adults [46,47,48,49], supporting its use in the current study.

A total of 24 items for the three components of TPB were all measured by using the Likert 5-point scale. The scale for intention ranges from easily possible (5) to impossible (1), and the scale for attitude, subjective norms, and PBC ranges from completely agree (5) to completely disagree (1). When the conceptual direction is opposite to the other items, the scale is set in the opposite direction. Reliability was verified to understand the internal consistency between the questionnaire items, and Cronbach’s α-coefficient was found to be 0.881 for attitude (6 questions), 0.913 for subjective norm (8 questions), and 0.819 for perceived behavioural control (4 questions).

#### 2.3.2. Food Literacy

Food literacy measure (origin, adaptation, and precedent in Chinese adults). We assessed food literacy using a comprehensive instrument originally developed and validated in Korea and organised into five domains—production, selection/choices, preparation and cooking, intake, and disposal—with 25 items rated on five-point Likert scales [52]. For use in the present study, the wording was adapted for mainland Chinese adults through forward translation, expert review, and a small pilot to ensure clarity and cultural appropriateness, while preserving the original domain structure and response anchors. Importantly, this Korean-developed scale has already been applied to Chinese adults in an independent study from Shandong Province, which reported graded increases in TPB constructs across food-literacy rank groups and a consistent, positive association of perceived behavioural control with healthy-eating intention after covariate adjustment—patterns that support the instrument’s construct validity in this population [53].

The five components of food literacy, the scale of production, selection, preparation and cooking, ingestion, and disposal, range from yes (4) to not at all (0). Reliability was verified to understand the internal consistency between the questionnaire items, and Cronbach’s α-coefficient was found to be 0.899 for production (7 questions), 0.832 for selection (5 questions), 0.873 for preparation and cooking (7 questions), 0.801 for intake (3 questions), and 0.812 for disposal (3 questions).

### 2.4. Other Variables

Demographic characteristics include gender (i.e., male and female), age (year), height (cm), weight (kg), monthly average income level (≤5000 CNY, 5,001–10,000 CNY, ≥10,001 CNY), alcohol intake within the year (no drinking experience, once, or more than one time), education level (i.e., high school or lower, university or higher), residence (i.e., first-tier cities, second-tier cities, third-tier cities, and other areas), disease diagnosed by a doctor (without and with), and the number of family members living together (person). Height and weight were used to calculate and distinguish the BMI (kg/m^2^), and the BMI categories were divided into <18.5 kg/m^2^, 18.5–22.99 kg/m^2^, 23.0–24.99 kg/m^2^, and >25.0 kg/m^2^ [51]. This standard differs from the World Health Organization (WHO) criteria, considering that the Chinese standard adopts a stricter definition of overweight and obesity to better align with the health characteristics of the Asian population.

### 2.5. Instrument Adaptation, Reliability, and Validity

Questionnaires were translated and culturally adapted by a bilingual expert panel; a pilot with nutrition-major students confirmed comprehensibility. Internal consistency (Cronbach’s α) was acceptable across constructs (e.g., attitude α = 0.881; subjective norms α = 0.913; PBC α = 0.819; intention α = 0.840; food-literacy domains α ≈ 0.80–0.90; and overall FL α = 0.957). Confirmatory factor analysis (CFA) supported the intended factor structures for TPB and food-literacy measures (global fit indices reported in the Results: e.g., CFI/IFI > 0.90; RMSEA ≈ 0.06; and SRMR < 0.05). Convergent validity was evaluated via standardised loadings, composite reliability (CR), and average variance extracted (AVE).

### 2.6. Statistical Analysis

We first screened the data for completeness and quality and used complete-case analysis. Descriptive statistics summarised participant characteristics and scale scores (mean ± SD; *n*, %). Group differences in behavioural intention were examined with independent-samples *t*-tests (binary factors) and one-way ANOVA (multi-level factors), reporting t/F, df, and *p*.

Measurement properties were assessed via confirmatory factor analysis (CFA) for TPB and food-literacy constructs; we report standardised loadings, CR/AVE, and global fit indices (e.g., CFI/TLI/IFI, RMSEA with 90% CI, SRMR). Internal consistency used Cronbach’s α.

To test moderation, we fit linear models including main effects (attitude, subjective norms, PBC, food-literacy domain[s]) and interaction terms (e.g., PBC × intake), adjusting for age, marital status, occupation, alcohol use, living with family, and physician-diagnosed chronic disease. Because VIFs indicated multicollinearity among TPB predictors and interactions, we used ridge regression (L2-regularised linear models) with standardised, mean-centred continuous variables and an unpenalised intercept. The penalty (λ) was selected by 10-fold cross-validation and inspection of the ridge trace; the final model used λ = 0.02 with CV-RMSE = 0.483 and CV-R^2^ = 0.631. We report unstandardised B, standardised β, 95% CIs, and *p*-values. Analyses were conducted in SPSS(29)/AMO(26) (descriptives, CFA) and R (glmnet) for ridge models.

## 3. Results

### 3.1. General Characteristics of Survey Subjects

The demographic characteristics of the 1145 subjects are summarized in Table 1. The sex distribution was nearly equal, with men accounting for 50.9% (*n* = 583) of the sample. Subjects aged 18–29 years comprised 35.8% of the study population. The proportions of underweight, overweight, and obese individuals were 14.0%, 18.7%, and 9.3%, respectively. Nearly half of the subjects (46.9%) reported an average monthly income of less than CNY 5000. Additionally, 48.0% indicated that they had not consumed alcohol during the previous year. More than half of the respondents (57.9%) had a university-level education or higher, and the majority resided in urban areas (Table 1).

### 3.2. Exploratory and Confirmatory Factor Analysis of the TPB and Food Literacy

Measurement model. Treating the Likert responses as ordinal and estimating with WLSMV on polychoric correlations, the TPB four-factor model showed good fit: χ^2^(203) = 1001.06, χ^2^/df = 4.93, CFI = 0.950, TLI = 0.943, RMSEA = 0.059 (90% CI: 0.055–0.062), and SRMR = 0.033. The five-factor food literacy model demonstrated acceptable fit: χ^2^(265) = 1420.64, χ^2^/df = 5.36, CFI = 0.931, TLI = 0.922, RMSEA = 0.062 (90% CI: 0.059–0.065), and SRMR = 0.042. Given the known sensitivity of χ^2^ to large samples, the incremental (CFI/TLI) and residual-based (RMSEA/SRMR) indices collectively support adequate model performance.

Standardised factor loadings ranged 0.657–0.808 across TPB constructs and 0.621–0.795 across food-literacy domains. Composite reliability was 0.799–0.914, with Cronbach’s α = 0.801–0.913. AVE met the 0.50 criterion for all constructs except choices (AVE = 0.496, borderline); however, its loadings and CR = 0.831, together with overall model fit, suggest acceptable convergent validity. Item means indicated generally favourable responses: TPB construct means 3.82–3.96 (on 1–5 scales) and food-literacy domain means 3.46–3.67 (overall food literacy is 3.58 ± 0.51 on 0–4 scales). Taken together, these results indicate reliable measurement and acceptable validity, providing a sound basis for subsequent structural and moderation analyses (Table 2).

### 3.3. Correlation Between the Basic Characteristics of Survey Subjects and Healthy Dietary Behaviour Intentions

As shown in Table 3, basic demographic and lifestyle characteristics, including age, marital status, occupation, alcohol intake, health status, and cohabitation with family members, were found to influence intentions to engage in healthy-eating behaviours. Future studies should account for the effects of these factors when analysing behavioural intentions to derive more precise and reliable results.

### 3.4. The Relationship Between the Three Structures of the TPB and Healthy-Eating Intentions

Table 4 presents the relationships between attitudes, subjective norms, PBC, and healthy-eating intentions within the TPB, accounting for basic characteristics such as age, marital status, occupation, alcohol intake, health status, and cohabitation with family members. Multiple regression analysis indicated that subjective norms and PBC were positively associated with healthy-eating intentions (B = 0.224, *p* = 0.000 and B = 0.612, *p* = 0.000), whereas attitude did not have a significant effect. The model explained 67.2% of the variance in healthy-eating intentions.

### 3.5. The Moderating Effect of Food Literacy on the Relationship Between the Components of the TPB and the Intention of Healthy Behaviour

Table 5 presents the moderating effect of food literacy on the relationships between the components of the TPB and healthy-eating intentions. Since the *p*-values for all interaction terms exceeded 0.05, food literacy did not exhibit a significant moderating effect on these relationships.

### 3.6. The Moderating Effect of Food Literacy Components on the Relationship Between TPB Components and Healthy-Eating Intentions

The results presented in Table 6 indicate that the production, selection, preparation and cooking, and disposal components of food literacy did not have a significant moderating effect on the relationships between the components of the TPB and healthy-eating intentions. In contrast, the intake component demonstrated a positive moderating effect on these relationships (B = 0.03, *p* < 0.001; B = 0.03, *p* = 0.001; and B = 0.02, *p* = 0.013).

### 3.7. Ridge Regression Tests of Intake Moderation on TPB–Intention Associations

Ridge moderation model. Because the TPB predictors and their interactions exhibited multicollinearity, we estimated an L2-regularised (ridge) linear model with ten-fold cross-validation. The model achieved CV-RMSE = 0.483 and CV-R^2^ = 0.631, indicating good out-of-sample explanatory power on the 1–5 intention scale (Table 7). We selected λ = 0.02 as the primary penalty based on the lowest cross-validated error and stabilisation of the ridge trace.

As shown in Table 8, subjective norms (Std. β = 0.169, *p* < 0.001) and perceived behavioural control (PBC; Std. β = 0.459, *p* < 0.001) were positive, statistically significant predictors of healthy-eating intention, whereas attitude was not (Std. β = 0.013, *p* = 0.66). Intake literacy’s main effect was small and non-significant (B = −0.002, *p* = 0.148). Critically, only the PBC × intake literacy interaction was significant (Std. β = 0.182, *p* < 0.001); the ATT × intake and SN × intake interactions were non-significant (*p* = 0.252 and 0.209, respectively). This pattern indicates that higher intake literacy strengthens the positive association between PBC and intention, consistent with the view that capability-related skills enhance an individual’s perceived control and translate more strongly into intention. All continuous predictors were mean-centred and standardised, controls (age, marital status, occupation, alcohol use, living with family, and physician-diagnosed chronic disease) were included, and the intercept was not penalised. Given ridge shrinkage, coefficient magnitudes are conservative; inference therefore emphasises the direction and relative size of effects alongside cross-validated performance.

Consistent with H1, PBC and subjective norms—but not attitude—emerged as the most salient correlates of intention in this sample; H2 (overall literacy moderation) was not supported. Exploratory domain-level analyses identified a single robust moderation effect for consumption literacy on the PBC → intention pathway, suggesting that skills directly tied to daily eating practices may amplify the translation of perceived control into intention.

## 4. Discussion

To address multicollinearity among Theory of Planned Behaviour (TPB) predictors and interaction terms, we estimated an L2-regularised ridge model with ten-fold cross-validation (λ = 0.02; CV-RMSE = 0.483; and CV-R^2^ = 0.631; Table 7 and Table 8). Subjective norms and perceived behavioural control (PBC) were robust positive correlates of healthy-eating intention, whereas attitude was not significant. Of the five food-literacy domains, only intake (intake) moderated a TPB pathway: the significant PBC × intake interaction indicates that higher intake literacy strengthens the link between perceived control and intention.

The absence of a significant attitude–intention association is coherent with TPB theory and meta-analytic evidence showing that, in health contexts, norms and PBC can exert more proximal influence on intentions than attitudes [39,44,54,55,56]. In a cultural milieu where interpersonal obligations (family, peers, and authorities) are salient and where time, cost, availability, and skills constrain action, normative pressure and perceived control may outweigh personal evaluations. Attitudes towards healthy eating were also high on average (≈3.8–4.0/5), suggesting restricted variance that could attenuate predictive power, even after regularisation.

Interpreted through a Motivation–Opportunity–Capability lens, food literacy functions as capability: knowledge and skills both facilitate behaviour directly and condition the strength of intention pathways [44,54]. Among the literacy domains, intake comprises immediately actionable competencies—assembling a balanced meal, selecting options suited to one’s health needs and circumstances—that plausibly reduce execution barriers and enhance task-specific self-efficacy. This helps explain why intake, rather than more distal domains such as production, choices, preparation/cooking, or waste disposal, magnified the effect of PBC on intention within a near-term outcome frame (“eating healthily in the next two weeks”). Prior research is consistent with this mechanism: practical food skills, frequency of home cooking, and effective nutrition-label use are associated with healthier selections and better diet quality [16,33,57,58,59].

These findings fit within the international evidence on the TPB, which identifies norms and PBC as key correlates of intentions in health domains [39,55,56], and they extend capability-based accounts by showing that the domain closest to the focal act (intake) specifically conditions the PBC → intention path. The intake-specific moderation may also reflect China’s rapidly evolving food environment and widespread uptake of convenience and delivery foods, in which simple, fast and context-fitting skills are especially consequential for translating perceived control into intended action.

The results carry several practical implications. Programmes should prioritise intake-focused food-literacy training—menu planning, quick healthy swaps, balanced-plate templates, and 30 s label reading—in community and workplace settings. Campaigns should emphasise PBC and intake skills rather than attitude appeals alone, making healthy choices feasible and easy at the point of decision. Given high platform use, micro-lessons and just-in-time prompts embedded within ordering apps and canteen systems can scaffold healthier selections. Educational efforts should be paired with supportive choice architecture (defaults, salt-reduction prompts, and traffic-light front-of-pack labels) in line with international policy directions [60].

Methodologically, the study modelled the five components of food literacy separately and used ridge regression with cross-validation to estimate moderation under multicollinearity, reporting out-of-sample performance. Conceptually, it operationalised food literacy as a capability within the TPB and demonstrated that the domain nearest to behavioural execution—intake—moderates the PBC → intention pathway, offering a more granular account than studies relying on a single overall literacy score. Strengths include a large sample, validated measures with acceptable fit, and cross-validated estimates. Limitations include the cross-sectional design (preventing causal inference), possible seasonality (autumn–winter data collection), reliance on self-report, differing response scales for TPB (1–5) versus food literacy (0–4)—mitigated by centring/standardising in ridge—and high inter-factor correlations among TPB constructs, which raise discriminant validity caveats despite regularisation.

Future work should test intervention trials that raise intake-focused literacy and assess downstream effects on intentions and behaviours; employ longitudinal designs to establish temporal ordering; integrate digital food-literacy tasks that operate at the point of choice; and examine psychosocial mediators such as self-efficacy and cultural value orientations. Multi-site studies would help assess the cross-cultural generalisability of the attitude–norm–control balance and of intake-specific moderation [16,33,39,44,54,57,58,59,60].

## 5. Conclusions

In this cross-sectional survey of Chinese adults, perceived behavioural control and subjective norms—but not attitude—predicted healthy-eating intention. Among the five food-literacy domains, only Consumption (formerly “intake”) moderated the PBC→intention pathway, indicating that proximal, point-of-choice skills strengthen the translation of control beliefs into intention. These findings suggest that programmes should prioritise consumption-focused skills (e.g., quick healthy swaps, simple menu planning, rapid label use) alongside enabling choice architectures rather than attitude appeals alone. Limitations include the cross-sectional design, self-report measures and possible seasonal influences. Future work should test interventions targeting Consumption skills, use longitudinal designs, and evaluate digital, just-in-time supports at the point of choice, including assessment of cross-cultural generalisability.

## Figures and Tables

**Table 1:** General characteristics of the study subjects (*n* = 1145).

Variable	Category	*n* (%) or Mean ± SD
Sex	Male	562 (49.1)
	Female	583 (50.9)
Age, years	Mean ± SD	36.01 ± 11.73
	18–29	410 (35.8)
	30–39	323 (28.2)
	40–49	267 (23.3)
	50–64	145 (12.7)
BMI, kg/m^2^	Mean ± SD	23.04 ± 7.17
	<18.5	160 (14.0)
	18.5–22.9	664 (58.0)
	23.0–24.9	214 (18.7)
	≥25.0	107 (9.3)
Marital status	Married	707 (61.7)
Employment	Employed (on-the-job)	786 (67.8)
Education	≤High school	482 (42.1)
	College or higher (incl. technical college)	663 (57.9)
Place of residence	Metropolises	137 (12.0)
	Second-tier cities	224 (19.6)
	Tier-three and below (other medium/small cities)	419 (36.6)
	County/town, rural	365 (31.9)
Alcohol use (past year)	Yes	550 (48.1)
	≥Once	595 (52.0)
Chronic disease	Yes	221 (19.3)
Monthly income, CNY	≤5000	537 (46.9)
	5001–10,000	294 (25.7)
	≥10,001	314 (27.4)
Living with family	Yes	873 (76.2)

Abbreviations: BMI (body mass index) categories follow Chinese adult criteria; CNY, Renminbi; Place of residence. Metropolises (≥5 M); Tier-two (1–5 M); small and medium cities (<1 M, urban districts incl. county-level cities); county cities/rural (outside urban districts). Thresholds use built-up-area permanent resident population; definitions follow national standards. Notes: Percentages are calculated with *n* = 1145 and may not sum to 100% due to rounding.

**Table 2:** Exploratory and confirmatory factor analysis of the TPB and food literacy.

Construct/Domain (Cronbach’s α)	Item	CFA Loading (λ)	Mean ± SD
Attitude (α = 0.881)	A healthy diet is generally beneficial.	0.73	3.98 ± 0.96
	A healthy diet is generally useful.	0.73	4.00 ± 0.92
	A healthy diet is generally good.	0.75	4.00 ± 0.94
	A healthy diet is generally enjoyable.	0.77	3.98 ± 0.94
	A healthy diet is generally interesting.	0.737	3.89 ± 1.00
	A healthy diet is generally desirable.	0.752	3.95 ± 0.92
	Scale score (Attitude)		3.96 ± 0.75
	Summary: CR = 0.882; AVE = 0.555		
Subjective norms (α = 0.913)	Family members think I should eat healthily.	0.657	3.97 ± 0.95
	My friends think I should have a healthy diet.	0.808	3.86 ± 0.98
	My schoolmates and co-workers think I should eat healthily.	0.745	3.88 ± 0.92
	Experts (doctors, nutritionists, etc.) think I should eat healthily.	0.798	3.87 ± 0.96
	Government agencies think I should have a healthy diet.	0.743	3.81 ± 1.01
	Television programmes (including internet content) think I should have a healthy diet.	0.759	3.87 ± 0.93
	Newspapers and magazines (including internet content) think I should have a healthy diet.	0.789	3.84 ± 0.96
	Online information (blogs, YouTube, etc.) thinks I should have a healthy diet.	0.732	3.87 ± 0.95
	Scale score (Subjective norms)		3.87 ± 0.76
	Summary: CR = 0.914; AVE = 0.570		
Perceived behavioural control (α = 0.819)	I can try hard to eat healthily.	0.725	3.88 ± 0.97
	I am fully trained in a healthy diet.	0.774	3.84 ± 0.98
	I have enough time to practise a healthy diet.	0.686	3.84 ± 0.90
	I want to practise a healthy diet no matter what the difficulties may be.	0.731	3.74 ± 0.98
	Scale score (PBC)		3.82 ± 0.77
	Summary: CR = 0.820; AVE = 0.533		
Behavioural intention (α = 0.840)	I am willing to have a healthy meal within the next two weeks.	0.754	3.83 ± 0.96
	I want to have a healthy meal in the next two weeks.	0.749	3.83 ± 0.99
	I have a plan to have a healthy meal in the next two weeks.	0.775	3.85 ± 0.94
	I would like to recommend healthy meals to my friends, family, and co-workers.	0.738	3.84 ± 0.97
	Scale score (Intention)		3.82 ± 0.80
	Summary: CR = 0.841; AVE = 0.569		
Food literacy—production (α = 0.899)	I usually check the country of origin of food.	0.753	3.43 ± 1.02
	I usually check the GMO mark.	0.732	3.35 ± 1.08
	I usually check agri-food certification (organic, pesticide-free, etc.).	0.782	3.50 ± 1.03
	I can look up information about production (e.g., ‘Animal Welfare’ certification).	0.776	3.41 ± 1.10
	I usually choose food based on the nutrition facts label.	0.712	3.56 ± 1.03
	I usually check food ingredients in processed foods.	0.747	3.55 ± 0.98
	I know how food distribution affects the environment and society.	0.738	3.43 ± 1.06
	Scale score (Production)		3.46 ± 0.82
	Summary: CR = 0.899; AVE = 0.561		
Food literacy—choices (α = 0.832)	I can find various distribution methods (local food, direct sales, etc.).	0.769	3.38 ± 1.05
	I can buy food efficiently (saving money/time).	0.648	3.72 ± 0.93
	I can look up ways to judge food quality (taste, freshness, etc.).	0.683	3.67 ± 0.96
	I can decide if I need food by looking at advertisements.	0.697	3.57 ± 1.01
	If I have food/health questions, I can find accurate information.	0.72	3.44 ± 1.08
	Scale score (Choices)		3.56 ± 0.78
	Summary: CR = 0.831; AVE = 0.496		
Food literacy—preparation and cooking (α = 0.873)	I usually check the expiration date of food.	0.681	3.85 ± 0.99
	I can discuss pros/cons of Chinese culinary culture.	0.621	3.51 ± 1.05
	I cook/store food while considering food-poisoning risks.	0.725	3.71 ± 0.95
	I keep food in ways that maintain its quality.	0.746	3.65 ± 0.94
	I can judge hygiene from the preparation/cooking process.	0.787	3.54 ± 1.05
	I try to know correct information about food and health.	0.707	3.69 ± 0.97
	I can reflect on my diet and judge pros/cons of my habits.	0.681	3.73 ± 0.93
	Scale score (preparation and cooking)		3.67 ± 0.74
	Summary: CR = 0.875; AVE = 0.502		
Food literacy—intake (α = 0.801)	I can find foods/menus that fit my health and situation.	0.789	3.63 ± 1.00
	I can prepare a nutritionally balanced meal.	0.758	3.58 ± 1.10
	I try to eat a variety of food groups evenly.	0.716	3.67 ± 1.02
	Scale score (intake)		3.63 ± 0.85
	Summary: CR = 0.799; AVE = 0.570		
Food literacy—waste disposal (α = 0.812)	I try to reduce food waste.	0.683	3.62 ± 0.97
	I know correct packaging/separation for food waste.	0.723	3.61 ± 0.96
	I handle waste carefully, understanding environmental impact.	0.795	3.59 ± 0.96
	Scale score (waste disposal)		3.61 ± 0.80
	Summary: CR = 0.778; AVE = 0.540		
Food Literacy—overall (α = 0.957)	Scale score (overall FL)	—	3.58 ± 0.51

Notes. CFA loadings are standardised. “Scale score” rows report per-item means. TPB items: 1–5 scale; food literacy items: 0–4 scale. CR = composite reliability; AVE = average variance extracted.

**Table 3:** Correlation between basic characteristics of subjects and health behaviour intentions.

Variables	Category	Behavioural Intention		
		MEAN ± SD	T/F	*p*
Gender	Men	3.82 ± 0.78	−0.276	0.782
	Males	3.84 ± 0.80		
Age, years	18–29	3.61 ± 0.80	31.278	<0.001
	30–39	3.75 ± 0.81		
	40–49	4.11 ± 0.66		
	50–64	4.12 ± 0.69		
BMI, kg/m^2^	<18.5	3.85 ± 0.85	1.290	0.276
	18.5–24.0	3.81 ± 0.81		
	24.0–28.0	3.72 ± 0.72		
	≥28.0	3.72 ± 0.72		
Marital status	Married	3.93 ± 0.77	5.209	<0.001
	Others	3.68 ± 0.80		
Occupation	Employed (on-the-job)	3.87 ± 0.76	2.595	0.010
	Unemployed (housewife, student, etc.)	3.74 ± 0.85		
Education	≤High school	3.87 ± 0.81	1.264	0.207
	≥College/Technical college	3.81 ± 0.77		
Place of residence	Metropolises	3.73 ± 0.84	0.863	0.460
	Second-tier cities	3.83 ± 0.75		
	Tier-three and below (other medium/small cities)	3.85 ± 0.79		
	County/town, rural	3.85 ± 0.80		
Alcohol use (past year)	Never	3.92 ± 0.77	3.693	<0.001
	≥Once	3.75 ± 0.80		
Chronic disease	Yes	3.64 ± 0.86	−3.960	<0.001
	No	3.88 ± 0.77		
Monthly income, CNY	≤5000	3.85 ± 0.81	0.672	0.511
	5001–10,000	3.79 ± 0.77		
	≥10,001	3.85 ± 0.79		
Living with family	Yes	3.86 ± 0.78	2.426	0.016
	No	3.73 ± 0.81		

Notes. Values are per-item means (1–5); higher scores indicate stronger intention. Binary variables were compared using Welch’s *t*-test; multi-level variables used one-way ANOVA (Welch ANOVA when variances were unequal). *p* values to three decimals, with *p* < 0.001 reported when applicable. BMI categories follow Chinese adult criteria; CNY, Renminbi.

**Table 4:** Multiple linear regression of healthy-eating behavioural intentions on TPB constructs (with covariate adjustment; *n* = 1145).

Predictor	B	SE	β	95% CI for B	t	*p*-Value
Attitude	0.044	0.034	0.042	−0.022, 0.110	1.29	0.196
Subjective norm	0.224	0.037	0.212	0.151, 0.297	6.03	<0.001 ***
Perceived behavioural control (PBC)	0.612	0.029	0.594	0.556, 0.668	20.93	<0.001 ***

Model fit: F(9, 1135) = 260.68, *p* < 0.001; adjusted R^2^ = 0.672. Covariates (entered in the same block, coefficients not shown): age, marital status, occupation, alcohol use (past year), physician-diagnosed chronic disease, and living with family. Notes: Outcome = behavioural intention (per-item mean, 1–5). Estimates are from a single-step multiple linear regression (ordinary least squares) with all predictors entered simultaneously. B = unstandardised coefficient; β = standardised coefficient; SE = standard error; CI = confidence interval; t = test statistic. Two-tailed significance: *p* < 0.001 ***. PBC, perceived behavioural control.

**Table 5:** The regulatory role of food literacy on the relationship between the three elements of planned behaviour theory and the intentions for healthy-eating behaviour.

Independent Variable	Dependent Variables: Intention				
	B	SE	t	*p*	Model Fit
Attitude	0.503	0.03	16.629	0.000 **	F = 137.494
FL	0.015	0.001	10.887	0.000 **	*p*-value < 0.000
FL × Attitude	0.001	0.001	0.978	0.328	Adjusted R^2^ = 0.519
Subjective norm	0.589	0.029	20.651	0.000 **	F = 167.755
FL	0.012	0.001	9.598	0.000 **	*p*-value < 0.000
FL × Subjective norm	0.001	0.001	0.425	0.671	Adjusted R^2^ = 0.568
PBC	0.025	29.065	0.000 **	0.000 **	F = 249.098
FL	0.001	6.451	0.000 **	0.001 **	*p*-value < 0.000
FL × PBC	0.001	0.032	0.974	0.985	Adjusted R^2^ = 0.662

** *p* < 0. 01. FL, food literacy. Adjustments were based on the age, marital status, occupation, alcohol intake, illness, and cohabitation with family members.

**Table 6:** The moderating effects of the five components of food literacy on the relationships among the three constructs of the Theory of Planned Behaviour (TPB).

Predictor	Independent Variable	Dependent Variables: Intention				
		B	SE	t	*p*	Model Fit
Food production	Attitude	0.6	0.027	22.159	0.000 **	F = 128.141 *p*-value < 0.000 Adjusted R^2^ = 0.505
	Production	0.009	0.001	8.464	0.000 **	
	Production × Attitude	0.001	0.001	0.842	0.4	
	Subjective norm	0.667	0.025	26.263	0.000 **	F = 161.321 *p*-value < 0.000 Adjusted R^2^ = 0.559
	Production	0.008	0.001	7.981	0.000 **	
	Production × Subjective norm	0.001	0.001	0.264	0.792	
	PBC	0.777	0.022	34.68	0.000 **	F = 239.123 *p*-value < 0.000 Adjusted R^2^ = 0.653
	Production	0.003	0.001	3.239	0.001 **	
	Production × PBC	0.001	0.001	−0.002	0.999	
Food choices	Attitude	0.577	0.028	20.903	0.000 **	F = 124.877 *p*-value < 0.000 Adjusted R^2^ = 0.498
	Choices	0.009	0.001	7.908	0.000 **	
	Choices × Attitude	0.001	0.001	−0.183	0.855	
	Subjective norm	0.667	0.025	26.263	0.000 **	F = 158.774 *p*-value < 0.000 Adjusted R^2^ = 0.555
	Choices	0.008	0.001	7.981	0.000 **	
	Choices × Subjective norm	0.001	0.001	0.264	0.792	
	PBC	0.767	0.023	33.794	0.000 **	F = 240.012 *p*-value < 0.000 Adjusted R^2^ = 0.654
	Choices	0.003	0.001	3.608	0.000 **	
	Choices × PBC	0.001	0.001	−0.626	0.532	
Preparation and cooking	Attitude	0.53	0.031	17.269	0.000 **	F = 129.265 *p*-value < 0.000 Adjusted R^2^ = 0.507
	Preparation and cooking	0.012	0.001	8.979	0.000 **	
	Preparation and cooking × Attitude	0.001	0.001	1.556	0.12	
	Subjective norm	0.618	0.029	21.139	0.000 **	F = 157.093 *p*-value < 0.000 Adjusted R^2^ = 0.552
	Preparation and cooking	0.009	0.001	7.001	0.000 **	
	Preparation and cooking × Subjective norm	0.001	0.001	0.428	0.668	
	PBC	0.727	0.024	30.75	0.000 **	F = 249.321 *p*-value < 0.000 Adjusted R^2^ = 0.662
	Preparation and cooking	0.007	0.001	6.512	0.000 **	
	Preparation and cooking × PBC	0.001	0.001	0.461	0.645	
Food intake	Attitude	0.558	0.029	19.119	0.000 **	F = 131.134 *p*-value < 0.000 Adjusted R^2^ = 0.511
	Intake	0.01	0.001	9.222	0.000 **	
	Attitude × Intake	0.003	0.001	3.531	0.000 **	
	Subjective norm	0.644	0.027	23.404	0.000 **	F = 162.726 *p*-value < 0.000 Adjusted R^2^ = 0.561
	Intake	0.008	0.001	8.11	0.000 **	
	Intake × Subjective norm	0.003	0.001	3.472	0.001 **	
	PBC	0.744	0.023	32.333	0.000 **	F = 250.467 *p*-value < 0.000 Adjusted R^2^ = 0.663
	Intake	0.006	0.001	6.545	0.000 **	
	Intake × PBC	0.002	0.001	2.497	0.013 *	
Waste disposal	Attitude	0.579	0.027	21.141	0.000 **	F = 120.599 *p*-value < 0.000 Adjusted R^2^ = 0.486
	Disposal	0.009	0.001	8.634	0.000 **	
	Disposal × Attitude	0.001	0.001	1.587	0.113	
	Subjective norm	0.653	0.026	25.178	0.000 **	F = 127.900 *p*-value < 0.000 Adjusted R^2^ = 0.504
	Disposal	0.007	0.001	7.508	0.000 **	
	Disposal × Subjective norm	0.001	0.001	0.772	0.44	
	PBC	0.749	0.022	34.257	0.000 **	F = 246.276 *p*-value < 0.000 Adjusted R^2^ = 0.659
	Disposal	0.005	0.001	5.685	0.000 **	
	Disposal × PBC	0	0.001	−0.368	0.713	

Notes. Ordinary least squares (OLS) models were estimated separately for each food-literacy component and TPB predictor (attitude, subjective norm, PBC). Values are unstandardized coefficients, B, with standard errors in parentheses. Continuous variables were mean centred before forming interaction terms. Outcome: behavioural intention (per-item mean, 1–5; higher = stronger intention). Model fit shows adjusted R^2^ and omnibus F test (model *p* value). *p* values are reported to three decimals; values <0.001 shown as “<0.001”. Significance: * *p* < 0.05 and ** *p* < 0.01. Abbreviations: PBC, perceived behavioural control.

**Table 7:** Cross-validated performance of ridge models.

λ (Lambda)	Scheme	CV-RMSE	CV-R^2^
0.06	lambda.min	0.496	0.611
0.015	lambda.1se	0.511	0.592
0.02	Selected (primary)	0.483	0.631

Notes: Ten-fold cross-validation with out-of-fold predictions. The final penalty (λ = 0.02) was chosen based on the best cross-validated performance and coefficient stabilisation on the ridge trace. Abbreviation: CV, cross-validated.

**Table 8:** Ridge moderation model predicting behavioural intention (λ = 0.02; *n* = 1141).

Predictor	Unstd. B	SE	Std. β	95% CI for B	*p*-Value
Attitude (ATT)	0.014	0.032	0.013	–0.049, 0.077	0.66
Subjective norm (SN)	0.179	0.034	0.169	0.112, 0.246	<0.001 ***
Perceived behavioural control (PBC)	0.474	0.031	0.459	0.412, 0.536	<0.001 ***
Intake literacy (INT)	–0.002	0.002	–0.063	–0.006, 0.001	0.148
ATT × INT	0.000	0.000	0.045	0.000, 0.001	0.252
SN × INT	0.000	0.000	0.05	0.000, 0.001	0.209
PBC × INT	0.001	0.000	0.182	0.001, 0.001	<0.001 ***

Model performance: CV-RMSE = 0.483 and CV-R^2^ = 0.631 (ten-fold CV). Controls (included but not shown): age, marital status, occupation, alcohol use, living with family, and physician-diagnosed chronic disease. Specification: L2-regularised linear model (ridge); continuous predictors were mean-centred and standardised; and intercept not penalised. Outcome: behavioural intention. Significance codes: *p* < 0.001 ***.

## Data Availability

The data are not publicly available due to privacy, access to data can be requested from the corresponding author.
